# *FGFR3* Gene Alterations and Protein Expression in Paired TURBT and Radical Cystectomy Archival Specimens of Muscle-Invasive Bladder Cancer

**DOI:** 10.3390/ijms27198698

**Published:** 2026-09-29

**Authors:** Makito Miyake, Nobutaka Nishimura, Sayuri Ohnishi, Yuki Oda, Takuto Shimizu, Takuya Owari, Kota Iida, Akira Tachibana, Mitsuru Tomizawa, Kenta Ohnishi, Shunta Hori, Yosuke Morizawa, Daisuke Gotoh, Yasushi Nakai, Nobumichi Tanaka

**Affiliations:** 1 Department of Urology, Nara Medical University, 840 Shijo-cho, Kashihara 634-8522, Nara, Japan; 2 Department of Prostate Brachytherapy, Nara Medical University, 840 Shijo-cho, Kashihara 634-8522, Nara, Japan

**Keywords:** urothelial carcinoma, gene mutation, FGFR3, bladder cancer, immunohistochemical staining, chemotherapy

## Abstract

This study investigated whether neoadjuvant chemotherapy (NAC) influences the detectability of fibroblast growth factor receptor 3 gene alterations (*FGFR3*alt) in muscle-invasive bladder cancer (MIBC). Among 173 patients who underwent radical cystectomy (RC), 70 had paired transurethral resection of bladder tumor (TURBT) and RC archival specimens available for *FGFR3* RNA testing and immunohistochemical (IHC) analysis. *FGFR3*alt were detected in only six tumors (8.6%), and 13 RC specimens (19%) showed *RNA* test failure, predominantly in archival samples older than 10 years. The assay-success concordance of *FGFR3* RNA test results between paired TURBT and RC specimens was high (73%), although one NAC-treated patient with *FGFR3*alt TURBT tissue converted to *FGFR3*-negative status in the RC specimen. IHC analysis demonstrated significantly higher FGFR3 protein expression in *FGFR3*alt tumors and a moderate correlation between paired TURBT and RC specimens, irrespective of NAC exposure. Because *FGFR3* alterations were detected in only a small number of cases, the study was underpowered to evaluate whether NAC influences *FGFR3*alt detectability. These findings underscore the need for validation in larger, multi-institutional datasets and integrated molecular profiling to clarify the impact of chemotherapy on FGFR3-driven bladder cancer biology.

## 1. Introduction

The current standard treatment for muscle-invasive bladder cancer (MIBC) consists of neoadjuvant chemotherapy (NAC), with or without immune checkpoint inhibitors, followed by radical cystectomy (RC) and adjuvant therapy [1]. Despite these multimodal approaches, up to half of patients eventually develop locally advanced or metastatic urothelial carcinoma (la/mUC) [2]. The therapeutic landscape for la/mUC has expanded substantially with the introduction of antibody–drug conjugates (ADCs) [3] and the selective pan-fibroblast growth factor receptor (FGFR) inhibitor erdafitinib, which demonstrated clinical benefit in the THOR trials [4,5]. *FGFR3* alterations represent one of the most common genetic events in urothelial carcinoma, particularly in non-muscle-invasive disease, and are associated with distinct clinical and biological behavior. Prior foundational work has characterized the frequency, molecular consequences, and clinical significance of FGFR3 mutations in bladder cancer [6,7]. Identification of *FGFR3* gene alterations (*FGFR3*alt) has therefore become increasingly relevant for biomarker-guided therapy. Thus, it is essential to understand the potential factors affecting *FGFR* test results of tissue specimens, to guide testing strategies for *FGFR*alt in patients with la/mUC.

THOR cohorts 1 and 2 is a global phase 3 trial of erdafitinib as compared with chemotherapy or immune checkpoint inhibitor treatment in patients with la/mUC with susceptible *FGFR3/2*alt who had progression after previous treatments [4,5]. Of 8396 with available tumor samples from 8733 patients, 7293 (86.9%) had valid laboratory test results and the remaining 1103 (13.1%) had test failure. In clinical practice, transurethral resection of bladder tumor (TURBT) specimens obtained from treatment-naïve bladder tumor, RC specimens treated with or without NAC, and metastatic site specimens in limited cases are selectable for molecular testing. However, the influence of NAC on the detectability of FGFR3 alterations in RC specimens remains poorly understood. No established guidance is available regarding which specimen type is most appropriate for FGFR3 molecular testing in clinical practice.

Previous studies have shown that *FGFR3*alt are enriched in luminal papillary tumors, which generally exhibit favorable biology [8,9]. NAC, however, can induce molecular subtype shifts toward basal phenotypes [2,10], raising the possibility that chemotherapy may alter the representation or detectability of FGFR3-mutated clones. Our recent research confirmed a marked shift in the distribution of molecular subtypes after NAC, with 15 (43%) of 35 luminal subtype tumors in pre-NAC samples changing to basal subtype tumors in post-NAC samples [2], which was consistent with the results of earlier publication [10].

To address this knowledge gap, we investigated the suitability of NAC-treated RC specimens for *FGFR3* RNA testing and FGFR3 protein expression analysis. By examining paired TURBT and RC specimens, we aimed to determine whether NAC affects *FGFR3*alt detectability and to clarify the practical implications for specimen selection in the era of FGFR-targeted therapy.

## 2. Results

### 2.1. Gene Expression of FGFR3 and Its Major Ligands, FGF1 and FGF2, in UC Cell Lines

Based on morphological characteristics, the urothelial carcinoma cell lines used in this study were categorized into luminal-like (UROtsa, MGH-U3, UM-UC-14, KU-7, and HT-1376) and basal/squamous-like subtypes (T24, UM-UC-3, and J82). Direct sequencing from our previous work identified *FGFR3* mutations in UM-UC-14 (S249C), MGH-U3 (Y373C), and J82 (K650E) [11]. Western blotting confirmed FGFR3 protein expression in UM-UC-14 and MGH-U3, but not in J82 [12].

**Figure 1:**
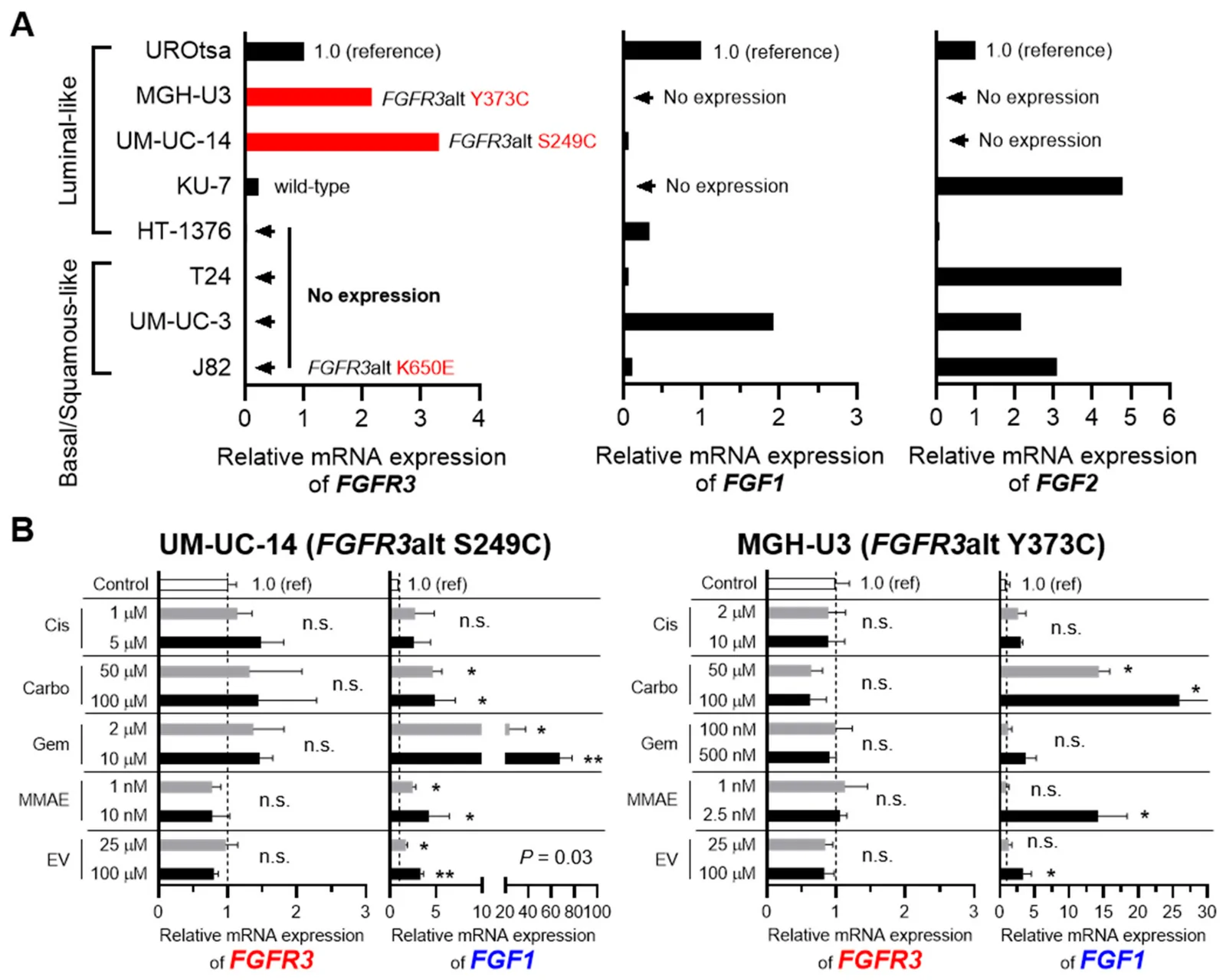
Chemotherapy-induced change in expression in *FGFR3* and *FGF1* genes in cell lines of urothelial carcinoma. (**A**) The mRNA expression level of *FGFR3*, *FGF1*, and *FGF2* in individual urothelial cancer cell lines is shown by relative value to UROtsa (immortal non-cancer urothelial cell). The mutations are indicated by red font. (**B**) UM-UC-14 and MGH-U3 cells have *FGFR3*alt S249C and *FGFR3*alt Y373C, respectively. These two cell lines are treated with cisplatin (Cis), carboplatin (Carbo), gemcitabine (Gem), MMAE, and EV at doses of IC50 and IC75 for 48 h. RNA gene expression level of *FGFR3* and *FGF1* is quantified with RT-PCR and shown by the relative value to non-treated control (set to 1.0). Data are expressed by mean ± standard deviation from three independent experiments. ‘FGFR3 (the receptor)’ and ‘FGF1 (the ligand)’ are emphasized by red and blue font, respectively. * *p* < 0.05, ** *p* < 0.01, n.s. not-significant.

Semiquantitative RT-PCR demonstrated 3.3-fold and 2.2-fold higher *FGFR3* expression in UM-UC-14 and MGH-U3, respectively, compared with UROtsa, whereas both cell lines showed minimal expression of *FGF1* and *FGF2* (Figure 1A). The expression levels are apparently different between luminal-like cells and basal/squamous-like cells, which is associated with phenotypic differences. Treatment of UM-UC-14 and MGH-U3 with cisplatin, carboplatin, gemcitabine, monomethyl auristatin E (MMAE), and enfortumab vedotin (EV) at IC50 and IC75 for 48 h did not alter *FGFR3* expression. In contrast, carboplatin, gemcitabine, MMAE, and EV increased *FGF1* expression (Figure 1B), while the *FGF2* expression level remained none to faint.

### 2.2. FGFR3 Gene Alteration in Paired TURBT and RC Specimens

**Figure 2:**
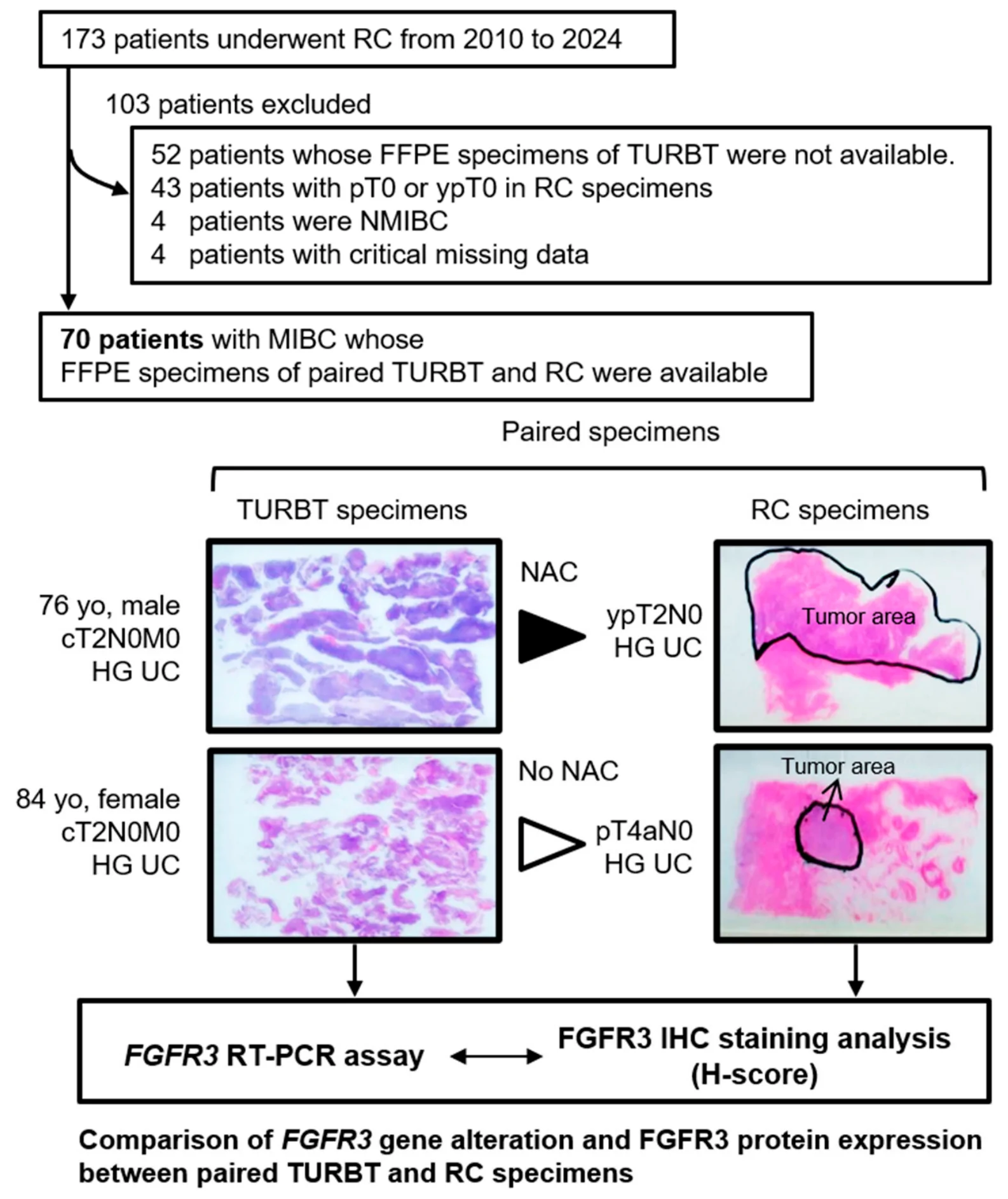
Flow chart showing the study design. Abbreviations: FFPE, formalin-fixed paraffin-embedded; IHC, immunohistochemistry; NAC, neoadjuvant chemotherapy; RC, radical cystectomy; RT-PCR, reverse transcription PCR; TURBT, transurethral resection; yo, years old.

Among 173 patients who underwent RC, 43 had pT0/ypT0 pathology at RC (Figure 2). These cases were excluded because no residual tumor was available for *FGFR3* RNA testing or FGFR3 immunohistochemical (IHC) analysis, which were required for paired molecular comparison. *FGFR3* testing of TURBT specimens was not performed in these patients, and baseline TURBT *FGFR3* status was unavailable. As a result, 70 met all inclusion criteria and had paired TURBT and RC specimens available for molecular testing (Figure 2). Among patients diagnosed with muscle-invasive disease (≥cT2) at initial diagnosis, a subset was found to have pTis or pT1 disease in the RC specimen (pathological downstaging), reflecting staging variability and tumor modification by TURBT or NAC.

**Figure 3:**
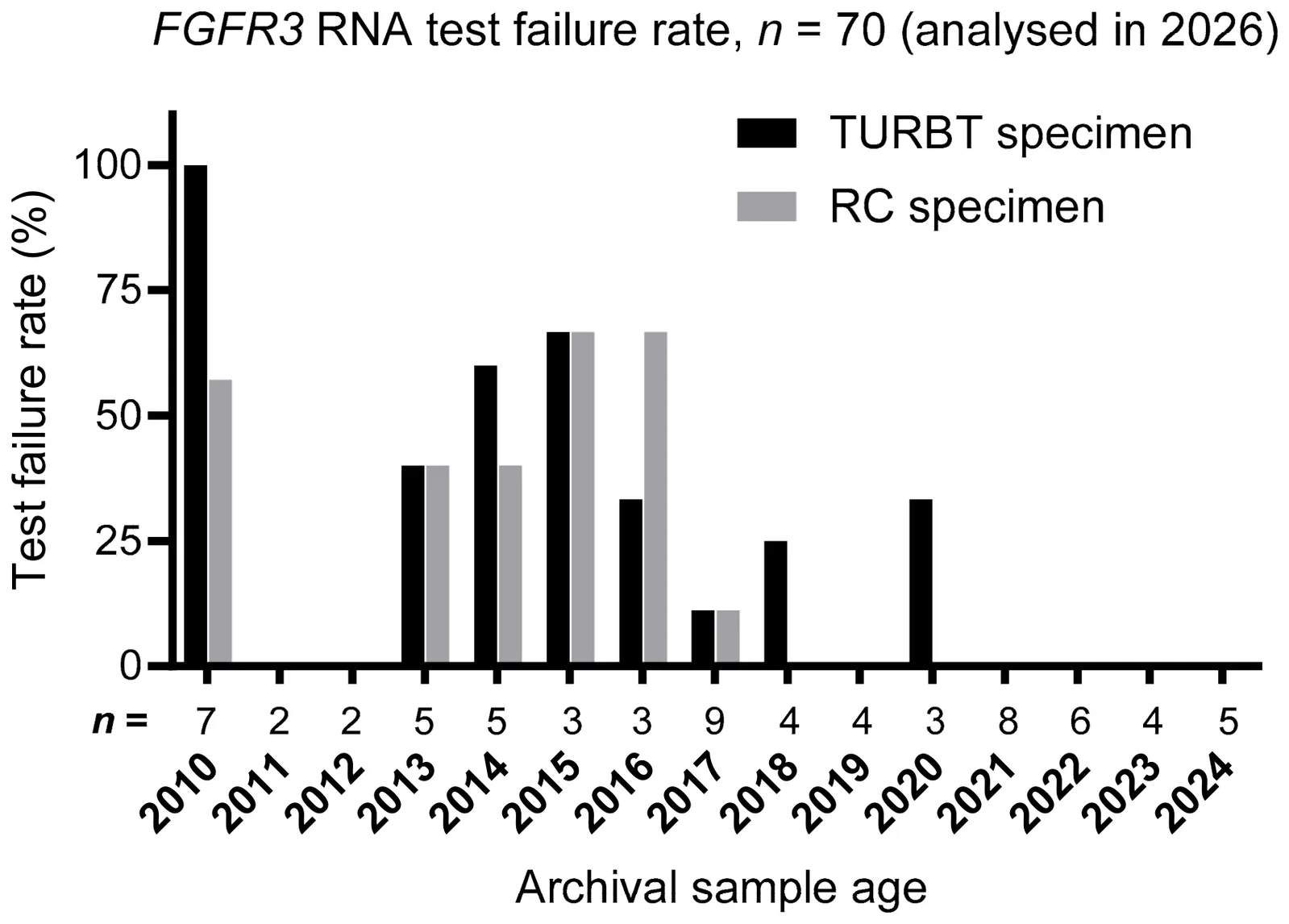
Association between *FGFR3* RNA test failure rates and the archival age of formalin-fixed paraffin-embedded (FFPE) specimens. Abbreviations: TURBT, transurethral resection; RC, radical cystectomy.

The characteristics of 70 patients with MIBC and comparison of the characteristics among test failure, *FGFR3*alt-negative, and *FGFR3*alt-positive cases are shown in Table 1. Of the 70 RC specimens, 13 (19%) showed test failure, predominantly in archival samples stored for ≥10 years. Figure 3 shows *FGFR3* test failure rates of 70 TURBT specimens and 70 RC specimens according to the archival sample age. No test failures occurred in RC specimens stored for ≤5 years (Fisher’s exact test, *p* = 0.044). Older formalin-fixed paraffin-embedded (FFPE) blocks, particularly those stored for >10 years, exhibited substantially higher RNA test failure rates in both TURBT and RC specimens, reflecting progressive RNA degradation over time. *FGFR3* alterations were detected in five RC specimens (7.1%), including S249C (*n* = 2) and Y373C (*n* = 3).

**Figure 4:**
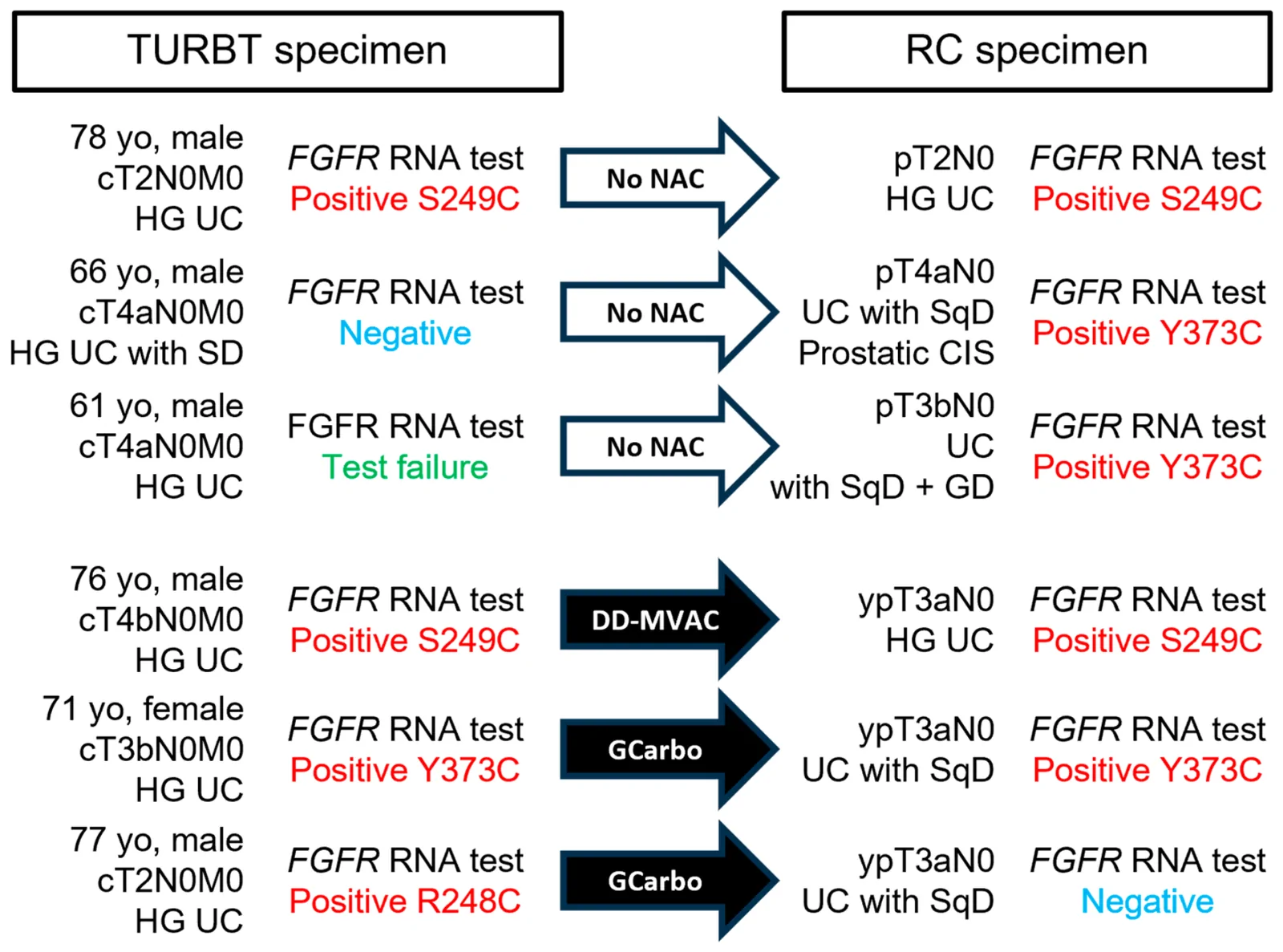
Details of six patients with FGFR3alt MIBC: comparison between paired TURBT specimens and RC specimens. Abbreviations: CIS, carcinoma in situ; DD-MVAC, dose-dense Methotrexate, Vinblastine, Doxorubicin, and Cisplatin combination chemotherapy; GCarbo, Gemcitabine and carboplatin combination chemotherapy; GD, glandular differentiation; HG, high-grade; UC, urothelial carcinoma; NAC, neoadjuvant chemotherapy; RC, radical cystectomy; SqD, squamous differentiation; TURBT, transurethral resection; yo, years old.

To avoid conflating technical assay performance with biological concordance, we separately evaluated assay-success concordance and *FGFR3* alteration-status concordance. Among the 70 paired TURBT–RC specimens, valid *FGFR3* RNA results were obtained for both specimens in 51 pairs (assay-success concordance: 73%). When restricting the analysis to these 51 evaluable pairs, FGFR3 alteration-status concordance was 96% (49/51). One NAC-treated patient with *FGFR3*alt-positive TURBT specimen had *FGFR3*alt-negative status in the RC specimen and another patient without NAC showed *FGFR3*alt-negative TURBT specimen and *FGFR3*alt-postive RC specimen (Figure 4). Pairs in which both specimens yielded invalid RNA results were excluded from the biological concordance calculation. Because *FGFR3* alterations were detected in only six patients, this concordance largely reflects agreement among alteration-negative cases. Concordance specifically among *FGFR3*-altered tumors could not be reliably assessed due to the very small number of altered cases.

### 2.3. FGFR3 Protein Expression in Paired TURBT and RC Specimens

**Figure 5:**
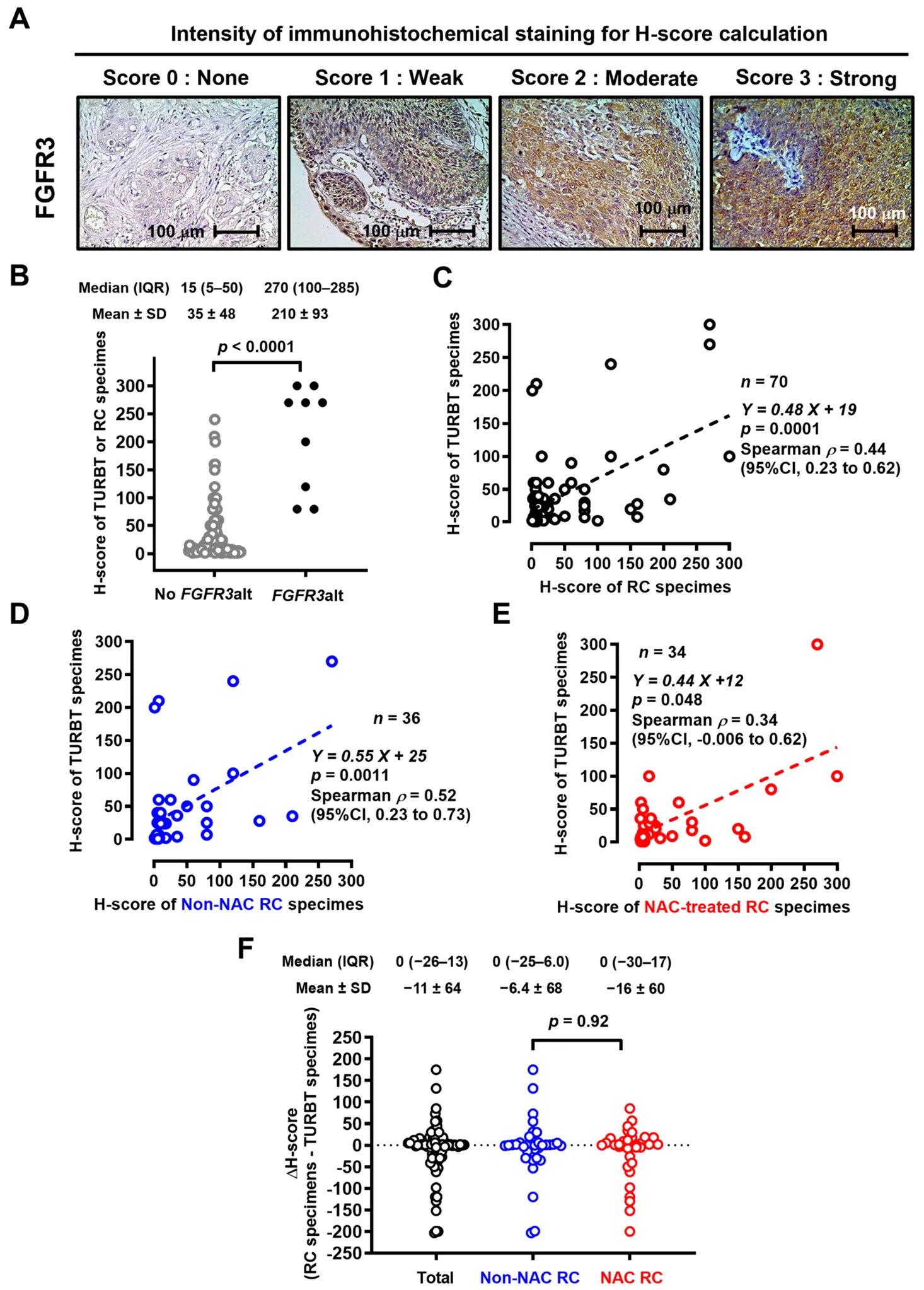
FGFR3 IHC staining analysis of paired TURBT specimens and RC specimens. (**A**) Representative images showing FGFR3 staining intensity scores of zero to three in patients who underwent RC. (**B**) FGFR3 H-scores of surgical specimens are presented using scatter plots and compared between patients without *FGFR3*alt and those with *FGFR3*alt. (**C**–**E**) Correlation of FGFR3 H-scores between TURBT specimens and paired RC specimens examined using Spearman’s correlation coefficient and linear regression analysis in total 70 cases (**C**), in 36 patients without NAC (**D**), and in 34 patients with NAC (**E**). (**F**) Paired changes in H-score were calculated as follows: ΔH-score = RC specimen H-score − TURBT specimen H-score. ΔH-scores were compared with Mann–Whitney *U* test. FGFR: fibroblast growth factor receptor; H-score: histochemical scoring method; RC: radical cystectomy; TURBT, transurethral resection.

FGFR3 IHC images with staining intensity scores of zero to three are shown as a reference (Figure 5A). FGFR3 IHC staining demonstrated significantly higher H-scores in *FGFR3*alt tumors compared with non-altered tumors (210 ± 93 vs. 35 ± 48; *p* < 0.0001) (Figure 5B). Correlation analysis revealed a moderate positive association between FGFR3 H-scores in paired TURBT and RC specimens (Spearman ρ = 0.44) (Figure 5C). The correlation was stronger in the non-NAC cohort (ρ = 0.52) than in the NAC-treated cohort (ρ = 0.34) (Figure 5D,E). NAC did not significantly affect FGFR3 protein expression in RC specimens compared with TURBT specimens (Wilcoxon signed-rank test, *p* = 0.64). To further assess whether NAC influences FGFR3 protein expression, we evaluated paired changes in H-score (ΔH-score = RC specimen H-score − TURBT specimen H-score) (Figure 5F). Among NAC-treated patients with evaluable paired IHC results, the median ΔH-score was 0 (interquartile range [IQR] −30–17), which was not significantly different from the ΔH-score observed in the non-NAC cohort (median 0, IQR −25–6.0; *p* = 0.92). Although the number of NAC-treated patients with paired IHC data was limited, no significant difference in FGFR3 expression was observed according to NAC exposure.

## 3. Discussion

This study explored whether neoadjuvant chemotherapy (NAC) influences the detectability of *FGFR3* alterations in MIBC using paired TURBT and RC specimens. Although the overall prevalence of *FGFR3* alterations was low (8.6%), several findings provide meaningful biological and practical insights into *FGFR3* testing in the NAC era.

A key observation was the high FGFR3 alteration-status concordance rate (96%) of *FGFR3* RNA test results between paired TURBT and RC specimens, indicating that FGFR3 alterations are generally stable across tumor compartments and surgical timepoints. This value must be interpreted cautiously. *FGFR3* alterations were uncommon in our cohort, and the *FGFR3* alteration-status concordance rate is driven predominantly by alteration-negative pairs. Therefore, the observed concordance does not necessarily indicate biological stability of *FGFR3*-altered clones across surgical timepoints. The study is underpowered to perform a meaningful agreement analysis focused on FGFR3-altered tumors, and larger datasets are required to evaluate concordance within this subgroup. This stability is consistent with prior reports showing that *FGFR3* mutations tend to be early clonal events in urothelial tumorigenesis and remain detectable across different tumor regions [10]. However, only one NAC-treated patient demonstrated loss of *FGFR3*alt in the RC specimen despite positivity in the TURBT specimen. While this discordance is of interest, the extremely small number of *FGFR3*-altered cases means that the present study is underpowered to determine whether NAC influences FGFR3 mutation detectability. Therefore, this finding should be considered hypothesis-generating only, and no inference regarding treatment-induced clonal selection can be made from our data.

Guercio et al. integrated prospectively collected clinical data with institutional genomic data to define the landscape of *FGFR2/3*-altered UC [13]. *FGFR3*alt alterations predictive of erdafitinib sensitivity were identified in 39% of patients with non-muscle-invasive disease, 14% with MIBC, 43% with localized upper tract UC, and 26% with metastatic UC specimens. Additionally, discordance of *FGFR3* mutational status between paired primary tumor and metastatic tumors occurred frequently (26%, seven out of 27 *FGFR3*alt cases) and raised potential concern over sequencing archival primary tumors to guide patient selection for erdafitinib treatment.

Another important finding was the marked impact of archival sample age on RNA test validity. RC specimens stored for ≥10 years exhibited significantly higher RNA test failure rates, consistent with the ANNAR biomarker study, which emphasized RNA degradation as a major determinant of FGFR test failure [14]. This reinforces the practical recommendation that recent FFPE blocks should be prioritized for FGFR testing, particularly when RNA-based assays are used. The association between archival sample age and RNA test failure observed in this study must be interpreted with caution. The number of specimens from individual years was small, and the use of broader archival categories was intended to stabilize descriptive trends rather than imply precise temporal thresholds. Furthermore, archival age may act as a surrogate for multiple preanalytical factors. Although our institution has maintained consistent formalin fixation protocols, minor changes in tissue-processing workflows, storage conditions, and block handling practices over the 2010–2024 period could also contribute to RNA degradation. Therefore, RNA failure cannot be attributed solely to archival age, and unmeasured preanalytical variability may confound this relationship.

The exclusion of 43 pT0/ypT0 cases introduces an inherent selection bias, as the NAC cohort analyzed in this study consists only of patients with residual tumor after treatment. Patients achieving ypT0 represent the most complete responders to NAC, yet they could not be included because no tumor tissue was available for *FGFR3* testing. Moreover, *FGFR3* status in these patients is unknown because *FGFR3* testing was not performed on their TURBT specimens. Therefore, our findings regarding NAC effects apply only to *FGFR3*-altered tumors with residual disease and may not be generalizable to the entire NAC-treated MIBC population.

FGFR3 protein expression demonstrated a clear biological association with *FGFR3*alt, as *FGFR3*-mutated tumors showed significantly higher H-scores than non-mutated tumors. This is in line with mechanistic studies showing that *FGFR3* missense mutations (e.g., S249C, Y373C) lead to ligand-independent receptor activation and protein overexpression [12]. Importantly, no significant difference in paired FGFR3 protein expression was observed according to NAC exposure, and paired TURBT–RC H-scores showed moderate correlation. However, this biological association should not be interpreted as evidence that FGFR3 IHC can serve as a clinical screening tool for FGFR-targeted therapy. FGFR3 protein overexpression is not equivalent to the presence of an actionable *FGFR3* mutation or fusion, and IHC cannot identify patients eligible for FGFR inhibitor treatment. Tumors classified as ‘FGFR3-negative’ may still harbor FGFR3 mutations or fusions not covered by the assay, and the FGFR3 alteration prevalence reported here reflects only alterations detectable by this specific platform. Our study was not designed to evaluate the diagnostic accuracy of FGFR3 IHC, and the findings should be interpreted strictly as biological correlations rather than clinical performance metrics. Therefore, FGFR3 IHC may provide supportive biological information when RNA testing fails, but it cannot substitute for RNA-based FGFR3 alteration testing in any therapeutic context.

This study has some limitations. First, the number of *FGFR3*-altered cases was small, limiting the statistical power to evaluate *FGFR3* alteration-status concordance and NAC-related effects specifically within *FGFR3*-altered tumors. Second, the retrospective single-center design may introduce institutional bias and restrict generalizability. Third, exclusion of pT0/ypT0 cases resulted in a selected cohort consisting only of patients with residual tumor, which may lead to selection bias when interpreting NAC-related findings. Fourth, the long study period (2010–2024) raises the possibility of temporal variation in preanalytical tissue-processing conditions, including fixation, processing, and storage, which may influence RNA quality independent of archival age. Fifth, tumor cellularity, sampling variability, and intratumoral heterogeneity may contribute to discordant RNA and IHC results despite efforts to use anatomically corresponding tumor areas and microdissection when necessary.

Taken together, our findings suggest that FGFR3 alterations are largely stable across TURBT and RC specimens, but NAC might influence detectability in a minority of cases. FGFR3 protein expression remains robust after NAC, supporting potential use as a complementary biomarker. Although the low *FGFR3*alt prevalence limited statistical power, this study provides practice-informing insights into specimen selection, archival age effects, and the biological behavior of FGFR3-driven tumors in the NAC setting. Larger, multi-institutional cohorts are needed to clarify the extent to which NAC modulates *FGFR3* molecular status and to refine testing strategies for FGFR-directed therapies.

## 4. Materials and Methods

### 4.1. Cell Lines

UROtsa (an SV40-immortalized non-transformed urothelial cell line without the characteristics of transformed cells) and seven UC cell lines were used in this study [2]. UROtsa, MGH-U3, KU-7, and J82 were obtained from Dr. Donald Sens (University of North Dakota School of Medicine, Grand Forks, ND, USA), Dr. H. LaRue (Laval University Cancer Research Centre, Québec, QC, Canada), Dr. Y. Nishiyama (Kyoto University, Kyoto, Japan), and Keio University, respectively. UM-UC-14 and HT-1376 were purchased from Sigma-Aldrich (St. Louis, MO, USA) and DS Pharma Bio-medical (Osaka, Japan), respectively. UM-UC-3 and T24 were purchased from ATCC (Manassas, VA, USA). All cell lines were maintained in DMEM or RPMI-1640 (Nacalai Tesque, Inc., Kyoto, Japan) supplemented with 10% fetal bovine serum (Nichirei Biosciences Inc., Tokyo, Japan), 100 U/mL penicillin, and 100 μg/mL streptomycin (FUJIFILM Wako Chemicals, Osaka, Japan) in a humidified incubator at 37 °C with 5% CO_2_.

### 4.2. Real-Time Reverse Transcription PCR (RT-PCR)

RNA extraction and real-time RT-PCR were performed as previously described [2]. TaqMan gene-specific primer/probe sets (Thermo Fisher Scientific, Applied Biosystems, Santa Clara, CA, USA) were used for *FGFR3* (Hs00179829_m1), *FGF1* (Hs01092738_m1), *FGF2* (Hs00266645_m1), and *GAPDH* (Hs02786624_g1), which served as the internal control. Relative mRNA expression was calculated using the standard curve method after normalization to *GAPDH*.

### 4.3. Antitumor Drugs

Gemcitabine, cisplatin, and carboplatin were purchased from Tokyo Chemical Industry (Tokyo, Japan). EV and MMAE were obtained from Selleck Chemicals (Houston, TX, USA). Stock solutions were prepared according to the manufacturers’ instructions.

### 4.4. Patient Selection and Data Collection

Medical records of 173 patients who underwent RC between January 2010 and December 2024 were reviewed. Clinicopathological data—including treatment period, age, sex, clinical/pathological stage, NAC implementation, and histological features—were collected. Inclusion criteria were (i) availability of FFPE tissue from both TURBT and paired RC specimens; (ii) presence of pathologically confirmed UC cells in both specimens. Patients with missing critical data, non-UC histology without any UC component, or pT0/ypT0 RC specimens were excluded. All hematoxylin and eosin (HE) slides of TURBT and RC were independently reviewed by experienced urologists, and staging was assigned according to the 8th edition of the UICC TNM classification [15]. HE slides were examined to confirm the presence of UC and to assess tumor cellularity. FFPE blocks containing sufficient UC cells were selected for *FGFR3* RNA testing and FGFR3 IHC analysis. When FFPE blocks contained limited tumor cellularity, manual microdissection was performed to enrich tumor content. Tumor-rich regions were carefully trimmed from unstained sections under microscopic guidance to ensure adequate tumor cellularity for RNA extraction. All procedures followed standard institutional pathology workflows.

In this cohort, NAC regimens consisted of cisplatin-based combinations (dose-dense MVAC or gemcitabine–cisplatin), carboplatin-based combinations (gemcitabine–carboplatin), and the other regimens. The other category included two patients treated with gemcitabine–paclitaxel and one patient treated with gemcitabine monotherapy, typically selected due to renal dysfunction or intolerance to platinum agents. Most patients received 2–4 cycles of NAC, depending on tolerability and clinical status.

### 4.5. RNA Testing for FGFR3 Alteration

Ten unstained 5 µm FFPE sections were used for RNA extraction with the RNeasy DSP FFPE kit (Qiagen, Hilden, Germany). *FGFR3* RNA testing was performed using the therascreen^®^ FGFR Rotor-Gene Q RT-PCR assay (Qiagen) through SRL, Inc. (Tokyo, Japan). The assay detects four *FGFR3* missense mutations (R248C, S249C, G370C, and Y373C) and two *FGFR3*::*TACC3* fusions (v1 and v3). The results were reported as *FGFR3*alt-positive, mutational type if positive, *FGFR3*alt-negative, or test failure due to undetectable amplification. To evaluate the association between archival age and RNA test failure, specimens were grouped into three predefined archival periods (1–5, 6–10, and 11–15 years). These categories were selected to provide adequate sample numbers within each group and to avoid over-interpreting year-specific fluctuations, as the number of specimens per individual year was small.

For each TURBT and RC specimen, *FGFR3* RNA testing and FGFR3 IHC were performed using the same FFPE tumor block. Unstained sections for RNA extraction and slides for IHC were prepared as serial sections to ensure anatomical correspondence of the sampled tumor area. When tumor cellularity within the block was limited, manual microdissection was performed to enrich tumor content for RNA extraction, and the same microdissected tumor region was used to guide selection of the corresponding area for IHC evaluation. This approach minimized the impact of intratumoral heterogeneity on molecular and protein expression analyses.

### 4.6. Immunohistochemical (IHC) Staining for FGFR3 Protein Expression

IHC staining was performed on FFPE blocks as previously described [2]. A mouse monoclonal anti-human FGFR3 antibody (clone B-9, sc-13121; Santa Cruz Biotechnology, Dallas, TX, USA) was used at a 1:50 dilution. FGFR3 expression was evaluated in three to five high-power fields (400×; 0.0625 mm^2^) of UC cells. The percentage of positively stained cells (1–100%) and staining intensity (0–3) were recorded. The histochemical score (H-score) was calculated as intensity × percentage of positive cells (0–300). Two investigators (Y. Oda and T. Shimizu), blinded to clinical data, independently assessed all slides. The H-score was determined by the average of two independent scores.

### 4.7. Statistical Analysis

Categorical variables were compared using the chi-square test or Fisher’s exact test. Continuous variables were expressed as mean ± standard deviation (SD) or median with IQR and compared using the Mann–Whitney U test. Correlations between FGFR3 H-scores in paired TURBT and RC specimens were evaluated using Spearman’s correlation coefficient (ρ) and linear regression (Y-slope). Correlation strength was defined as weak (ρ < 0.4), moderate (0.4–0.7), or strong (>0.7). The Wilcoxon signed-rank test was used to compare paired H-scores. All analyses were performed using GraphPad Prism version 10 (GraphPad Software, San Diego, CA, USA). A two-sided *p* value < 0.05 was considered statistically significant.

## 5. Conclusions

Given the very small number of *FGFR3*-altered tumors, the study was underpowered to assess whether NAC affects *FGFR3* alteration status. Any potential NAC-related effects observed in this cohort should be interpreted with caution. These findings underscore the need for validation in larger, multi-institutional datasets and integrated molecular profiling to clarify the impact of chemotherapy on FGFR3-driven bladder cancer biology.

## Figures and Tables

**Table 1:** Characteristics of 70 patients with muscle-invasive bladder cancer undergoing RC with or without neoadjuvant chemotherapy.

Variables		Total	*FGFR3* RNA Test of RC Specimen			
			Test Failure	No Alteration	Alteration	*p* Value #
No. of patients		70 (100%)	13 (19%)	52 (74%)	5 (7.1%)	-
Treatment period (archival sample age)	2010–2014 (15–11 yrs)	21 (30%)	8 (62%)	12 (23%)	1 (20%)	0.044 †
	2015–2019 (10–6 yrs)	23 (33%)	5 (38%)	16 (31%)	2 (40%)	
	2020–2024 (5–1 yrs)	26 (37%)	0	24 (46%)	2 (40%)	
Sex	Male	55 (79%)	11 (85%)	40 (77%)	4 (80%)	0.88 †
	Female	15 (21%)	2 (15%)	12 (23%)	1 (20%)	
Age at diagnosis of MIBC	(mean ± SD)	71 ± 10.5	70 ± 9.7	71 ± 11.0	70 ± 7.0	0.15 ‡
Clinical T category #	cT2	37 (53%)	8 (62%)	28 (54%)	1 (20%)	0.09 †
	cT3	19 (27%)	3 (23%)	15 (29%)	1 (20%)	
	cT4	14 (20%)	2 (15%)	9 (17%)	3 (60%)	
Clinical N category #	cN0	60 (86%)	10 (77%)	45 (87%)	5 (100%)	NA
	cN+	10 (14%)	3 (23%)	7 (13%)	0	
*FGFR3* RNA test of TURBT specimen	Test failure	18 (26%)	12 (92%)	5 (9.6%)	1 (20%)	<0.0001
	No alteration	48 (69%)	1 (7.7%)	46 (89%)	1 (20%)	
	Alteration	4 (5.7%)	0	1 (1.9%)	3 (60%)	
Neoadjuvant chemotherapy	No	36 (51%)	7 (54%)	26 (50%)	3 (60%)	0.65 †
	Cisplatin-based	22 (31%)	6 (46%)	15 (29%)	1 (20%)	
	Carboplatin-based	9 (13%)	0	8 (15%)	1 (20%)	
	Other	3 (4.3%)	0	3 (5.8%)	0	
Pathological T category #	pTis	8 (11%)	2 (15%)	6 (12%)	0	0.94 †
	pT1	7 (10%)	3 (23%)	4 (7.7%)	0	
	pT2	19 (27%)	3 (23%)	15 (29%)	1 (20%)	
	pT3	25 (36%)	3 (23%)	19 (37%)	3 (60%)	
	pT4	11 (16%)	2 (15%)	8 (15%)	1 (20%)	
Pathological N category #	pN0	45 (64%)	9 (69%)	34 (65%)	2 (40%)	0.12 †
	pN+	19 (27%)	4 (31%)	14 (27%)	1 (20%)	
	pNx (no LND)	6 (8.6%)	0	4 (7.7%)	2 (40%)	
CIS in specimens	No	40 (57%)	5 (39%)	30 (58%)	5 (100%)	NA
	Yes	30 (43%)	8 (61%)	22 (42%)	0	
Non-UC histology in specimens	No	43 (61%)	8 (62%)	33 (64%)	2 (40%)	0.37 †
	Yes	27 (39%)	5 (38%)	19 (36%)	3 (60%)	

CIS, carcinoma in situ; LND, lymphonode dissection; MIBC, muscle-invasive bladder cancer; NA, not available; RC, radical cystectomy; TURBT, transurethral resection of bladder tumor; SD, standard deviation; UC, urothelial carcinoma; #, 8th Edition of the AJCC Cancer Staging; †, Fisher’s exact test; ‡, Kruskal–Wallis test.

## Data Availability

Data are available on request due to restrictions.

## Abbreviations

MIBC	Muscle-invasive bladder cancer
NAC	Neoadjuvant chemotherapy
RC	Radical cystectomy
la/mUC	Advanced or metastatic urothelial carcinoma
ADC	Antibody–drug conjugates
FGFR	Fibroblast growth factor receptor
*FGFR3*alt	FGFR3 gene alteration
TURBT	Transurethral resection of bladder tumor
RT-PCR	Real-time reverse transcription PCR
EV	Enfortumab vedotin
MMAE	Monomethyl auristatin E
FFPE	Formalin-fixed paraffin-embedded
